# Network-based cancer genomic data integration for pattern discovery

**DOI:** 10.1186/s12863-021-01004-y

**Published:** 2021-12-10

**Authors:** Fangfang Zhu, Jiang Li, Juan Liu, Wenwen Min

**Affiliations:** 1 State Key Laboratory of Nuclear Resources and Environment and School of Water Resources and Environmental Engineering, East China University of Technology, Nanchang, 330013 China; 2 State Key Laboratory of Nuclear Resources and Environment and School of Chemistry, Biology and Materials Science, East China University of Technology, Nanchang, 330013 China; 3 School of Computer Science, Wuhan University, Wuhan, 430072 China; 4 School of Mathematics and Computer Science, Jiangxi Science and Technology Normal University, Nanchang, 330038 China; 5 Information School, Yunnan University, Kunming, 650091 China

**Keywords:** Gene co-expression analysis, Differentially co-expression analysis, Gene interaction network, Sparse SVD, Structured sparse learning

## Abstract

**Background:**

Since genes involved in the same biological modules usually present correlated expression profiles, lots of computational methods have been proposed to identify gene functional modules based on the expression profiles data. Recently, Sparse Singular Value Decomposition (SSVD) method has been proposed to bicluster gene expression data to identify gene modules. However, this model can only handle the gene expression data where no gene interaction information is integrated. Ignoring the prior gene interaction information may produce the identified gene modules hard to be biologically interpreted.

**Results:**

In this paper, we develop a Sparse Network-regularized SVD (SNSVD) method that integrates a prior gene interaction network from a protein protein interaction network and gene expression data to identify underlying gene functional modules. The results on a set of simulated data show that SNSVD is more effective than the traditional SVD-based methods. The further experiment results on real cancer genomic data show that most co-expressed modules are not only significantly enriched on GO/KEGG pathways, but also correspond to dense sub-networks in the prior gene interaction network. Besides, we also use our method to identify ten differentially co-expressed miRNA-gene modules by integrating matched miRNA and mRNA expression data of breast cancer from The Cancer Genome Atlas (TCGA). Several important breast cancer related miRNA-gene modules are discovered.

**Conclusions:**

All the results demonstrate that SNSVD can overcome the drawbacks of SSVD and capture more biologically relevant functional modules by incorporating a prior gene interaction network. These identified functional modules may provide a new perspective to understand the diagnostics, occurrence and progression of cancer.

## Background

With the rapid development of (single-cell) RNA-Seq and microarray technologies, huge number of cancer genomic data have been generated [1–3]. The data provide some new opportunities to study on the gene cooperative mechanisms [4–8]. Based on the hypothesis that genes with similar functions may show similar expression patterns, clustering techniques have been used to identify co-expressed gene sets in which genes present similar expression patterns across all samples. However, these traditional clustering techniques face with the limitation that some genes can co-regulate across some samples rather than all samples in the real biological systems [9]. Therefore, many biclustering methods [4, 10–13] are proposed to discover some co-expressed gene sets in which genes present similar expression patterns across some samples.

Recently, several Sparse Singular Value Decomposition (SSVD) based methods have been proposed for biclustering gene expression data to discover gene functional modules (biclusters) [14], such as ALSVD [4], L0SVD [15], and so on. However, most of them ignore the prior gene interaction network knowledge from a protein protein interaction (PPI) network, whereas such information is very useful to improve biological interpretability of discovered gene modules [16–18]. The PPI network has been used in many biological applications for accurate discovery or better biological interpretability [16, 19–22]. However, as far as we know, there is very little work to incorporate the gene interaction network knowledge from PPI network into the bi-clustering framework. To address it, we integrate the gene network in the SSVD model for biclustering gene expression [23].

**Fig. 1:**
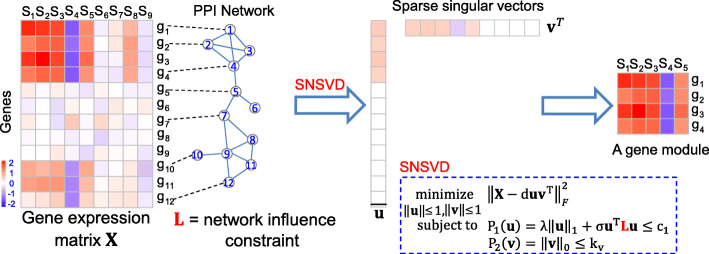
Overall workflow of Sparse Network-regularized Singular Value Decomposition (SNSVD). SNSVD integrates both a gene expression and a normalized Laplacian matrix ***L*** encoding a protein-protein interaction (PPI) network to identify gene functional modules. Based on the output of SNSVD (i.e., sparse singular vectors ***u*** and ***v***), we can identify a gene module whose members are from the nonzero elements of ***u*** and ***v***. Herein, we show a toy example to explain how SNSVD works. The gene module identified by SNSVD contains four genes (*g*_1_,*g*_2_,*g*_3_,*g*_4_) and five samples (*s*_1_,*s*_2_,*s*_3_,*s*_4_,*s*_5_), where the four genes are correlated across the five samples and the four genes correspond to a dense subnetwork of PPI network

In this paper, we develop a sparse network-regularized SVD (SNSVD) model to identify gene functional modules by integrating gene expression data and a prior gene interaction network from a PPI network (Fig. 1). To ensure the discovered gene modules in which genes are co-expressed and densely connected in the prior PPI network, we introduce a sparse network-regularized penalty [20] in the model. Compared with the traditional regularized penalties (e.g., LASSO [24]), the sparse network-regularized penalty can make the biclustering process tend to select correlated and interacted genes for enhancing biological interpretability of gene modules. We present an alternating iterative algorithm to solve the SNSVD model. We evaluate the performance of SNSVD using a set of artificial data sets and gene expression data from the Cancer Genome Project (CGP) [3], and compare its performance with other representative SSVD methods. We investigate the functionality of these identified modules from multiple perspectives. The results show that SNSVD can identify more biologically relevant gene sets and improve their biological interpretations.

Additionally, we present a framework based on SNSVD for analyzing matched miRNA and mRNA expression data to identify differentially co-expressed miRNA-gene modules. Extensive results when applying onto TCGA breast cancer data demonstrate that the identified miRNA-gene modules provide a new perspective for diagnosis and discrimination between two status of breast cancer.

## Results

### Simulation study

We evaluated the performance of SNSVD on the simulated data by comparing it with other sparse SVD based methods including L0SVD [15], ALSVD [4] and SCADSVD [4, 25]. Without loss of generality, we define a rank-one true signal matrix as ***uv***^*T*^ where ***u*** and ***v*** are vectors of size *p*×1 and *n*×1, respectively. The observed matrix is defined as ***X***=***uv***^*T*^+*γ****ε***, where ***ε*** is a noise matrix each element in which is randomly sampled from a standard normal distribution and *γ* is a nonnegative parameter to control the signal-to-noise ratio (SNR).

**Fig. 2:**
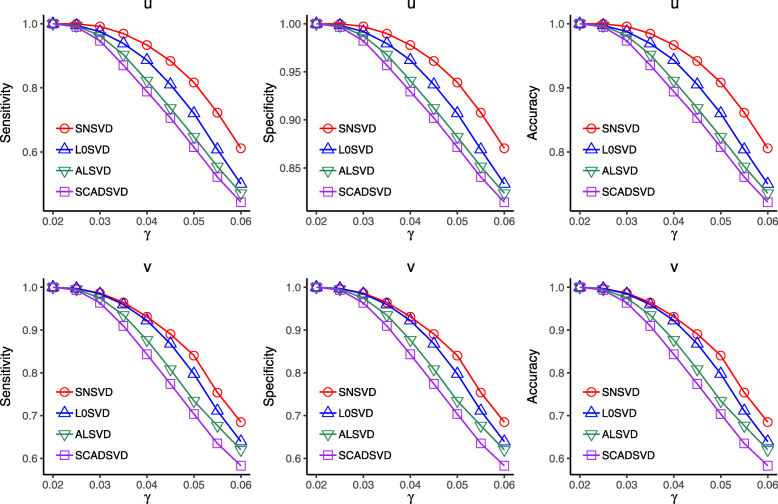
Performance of different methods on simulated data when *γ* is varied (*γ* is a parameter to control the signal-to-noise ratio). “Sensitivity” denotes the percentage of true non-zero entries in the identified vector, “Specificity” denotes the percentage of true zero entries in the identified vector, and “Accuracy” denotes classification accuracy

To generate the simulated data, we first generated two sparse singular vectors ***u*** and ***v*** with *p*=200,*n*=100 whose first 50 elements equal to 1 (non-zeros), and the remaining ones are zeros. Then we created a series of observation matrices ***X*** for each *γ* ranging from 0.02 to 0.06 in steps of 0.005. In addition, we created a prior “PPI” network for row variables of ***X***, where any two nodes in first 50 vertices are connected with probability *p*_11_=0.3, and remaining ones are connected with probability *p*_12_=0.1. For each *γ*, we generated 50 different noise matrices ***ε*** to got 50 observed matrices ***X*** for testing. The average sensitivity, specificity and accuracy of ***u*** (or ***v***) on the 50 matrices ***X*** were calculated. Moreover, we set *σ*=0.5 according to 5-fold cross validation test, and forced ***u*** and ***v*** to contain 50 non-zero elements with same sparsity level for each method by tuning the parameters so that the results of different methods are comparable. The average sensitivities, specificities and accuracies of ***u*** (or ***v***) with different *γ* were compared in Fig. 2. We found that the performance of our proposed method (SNSVD) is superior to that of other methods. The results illustrate that SNSVD model can enhance the power of variable selection by integrating the prior network knowledge.

### Application to the CGP gene expression data sets

We further investigated the performance of SNSVD by using the gene expression data with 641 cell lines including diverse cancer types and tissues from the Cancer Genome Project (CGP) (http://www.cancerrxgene.org/downloads) [3], and a PPI network from the Pathway-Commons database [26]. In total, there are 13,321 genes and 262,462 interactions in the PPI network. The 641 cell lines are from 16 tissues or 52 cancer types in the CGP data, where a tissue type contains about 40 cell lines and a cancer type contains about 12 cell lines.

#### Identifying functional modules

**Fig. 3:**
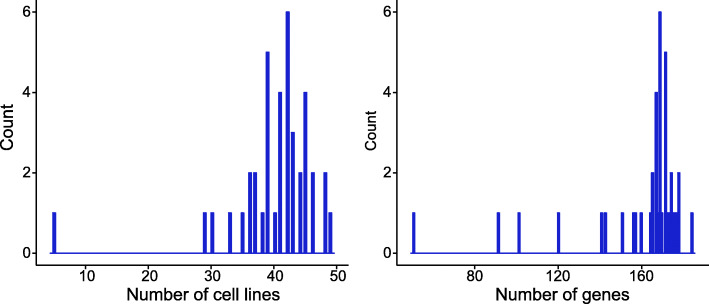
Distribution of the number of samples (i.e., cell lines) and genes from the identified modules by SNSVD on the CGP data

We set *σ*=100 according to 5-fold cross validation test, and set *k*_*v*_=50 (control the sample sparsity). In addition, we also selected a suitable *λ* to force the estimated ***u*** only containing 200 nonzero elements (control the gene sparsity). Using Algorithm 3, we identified the first 40 pairs of singular vectors {(***u***_1_,***v***_1_),⋯,(***u***_40_,***v***_40_)}. Let ***U***=[***u***_1_;⋯ ;***u***_40_] and ***V***=[***v***_1_;⋯ ;***v***_40_], where the *i*th column of ***U*** and ***V*** correspond to the *i*th pair of sparse singular vectors. To reduce the false positive cases, we first calculated absolute z-score for each column of ***U*** (or ***V***) according to Eq. (1). For each non-zero *x*_*ij*_, we define the following formula:

1 $$ z_{{ij}} = \frac{||x_{{ij}}|-\mu_{j}|}{\sigma_{j}},  $$

where *x*_*ij*_ is *i*-th element in ***u***_*j*_ (or ***v***_*j*_), *μ*_*j*_ is the average value of all non-zero elements in ***u***_*j*_ (or ***v***_*j*_), and *σ*_*j*_ is their standard deviation. If *z*_*ij*_ is greater than a given threshold, the corresponding gene (or sample) is then selected into the module *j*. Herein, we obtained 40 gene functional modules with 160 genes and 40 samples in average (Fig. 3).

#### Functional analysis of the genes in modules

**Fig. 4:**
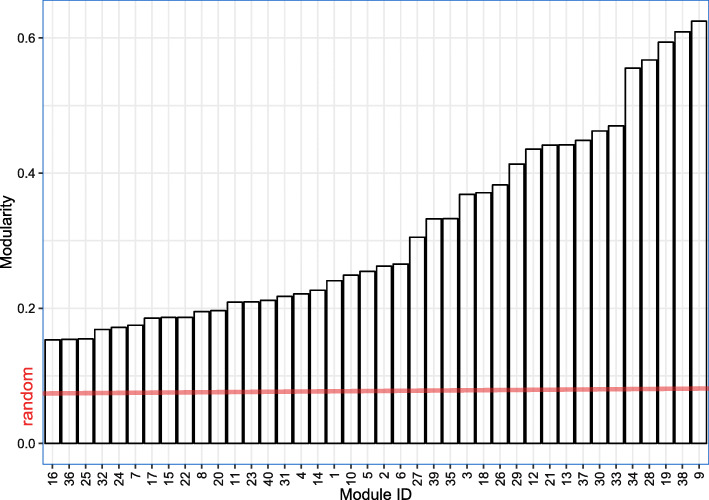
The modularity scores of the identified modules by SNSVD on the CGP data. The red line in plot denotes an average modularity score of randomized modules (gene sets)

Firstly, we investigated whether the genes within the same modules are significantly co-expressed by calculating the modularity score in Eq. (16), the result showed that all identified modules were statistically significant with *p*-value <0.01 by using one-sided Wilcoxon signed rank test (Fig. 4).

Secondly, we also investigated whether the genes within the same modules are connected with each other in the prior PPI network via the gene-gene interaction enrichment score. The result showed that 57% of the 40 modules were significantly inter-connected with each other in the PPI network, illustrating that our method tend to cluster genes interacting with each other.

Finally, we also checked the biological relevance of all the identified gene modules using gene functional enrichment analysis via DAVID online web tool [27]. By selecting the GO BP (Gene Ontology Biological Process) and KEGG pathways with Benjamini-Hochberg adjusted *p*-values<0.05 as significant ones, we obtained 766 significant GO BP pathways and 70 significant KEGG pathways. By statistically, 62.5% modules are significantly related with at least one GO BP pathways and 42.5% modules are significantly related with at least one KEGG pathways.

#### Functional analysis of the samples in modules

**Fig. 5:**
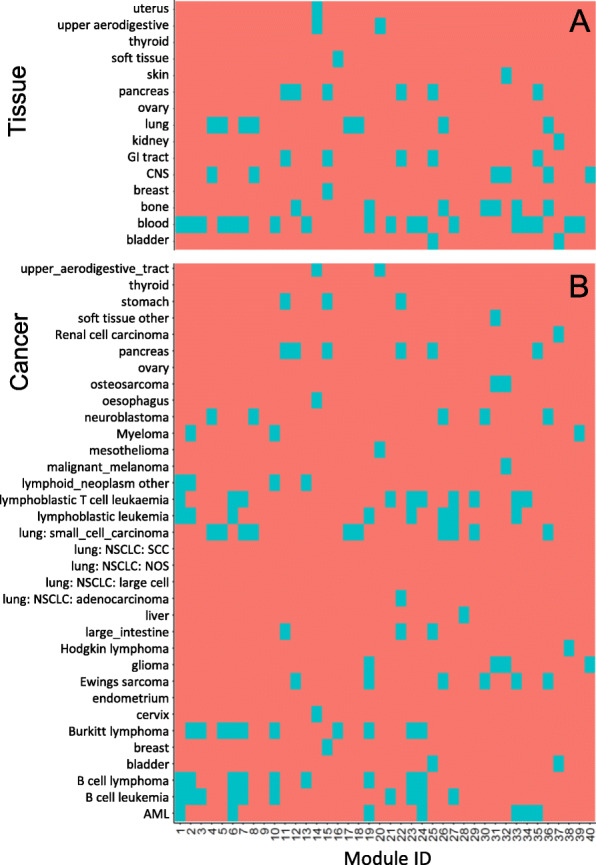
These identified gene modules by SNSVD are subtype-specific related to some tissues or cancer types. **A** is a heatmap in term of different tissues. **B** is a heatmap in term of different cancer types. Note that each blue square in the two heatmaps corresponds to a significance overlapping relationship (Hypergeometric test, *p* <0.05)

To evaluate the subtype-specific of samples in the identified modules, we computed the overlapping significance level of between module-samples and cancer/tissue specific samples. For each gene module, we first collected a sample set from the module. We then computed the overlapping significance levels between the sample set and any one tissue-sample set using the right hypergeometric test (Fig. 5A), and the overlapping significance levels between the sample set and any one cancer-sample set (Fig. 5B). We found that most of the identified gene modules can be seen as subtype-specific gene functional modules, which provide insights into the mechanisms of the relationship between different tissues and cancers.

Additionally, we also found that the cancer/tissue types of some modules are consistent with their corresponding functional pathways. Some examples are listed in Table 1 (See also Fig. 5). For example, module 1 contains 47 cell lines significantly overlapping with blood tissue and some blood-related cancers (e.g., AML, B cell leukemia, B cell lymphoma, lymphoblastic leukemia, lymphoblastic T cell leukaemia, lymphoid_neoplasm other), while the top enriched GO/KEGG pathways of 174 genes in module 1 are related to the immune system. Some previous work have reported that the development of blood-related cancers are associated with immune pathway abnormalities [28, 29]. Similarly, these samples in module 2 are also significantly related with some blood-related cancers (B cell leukemia, B cell lymphoma, Burkitt lymphoma, lymphoblastic leukemia, and lymphoid_neoplasm other), while some genes in which are significantly enriched in some immune-related pathways. These samples in module 4 are significantly related with central nervous system (CNS), while some genes in which are significantly enriched in nervous system related GO/KEGG pathways.

**Table 1:** The first five enriched GO/KEGG pathways of top ten modules identified by SNSVD on the CGP data where “*P*-value” denotes Benjamini-Hochberg adjusted *P*-value

Module	Enriched GO/KEGG pathways	*P*-value
1	GO:0006952˜defense response	3.08e-12
1	GO:0001775˜cell activation	1.11e-10
1	GO:0045321˜leukocyte activation	2.55e-10
1	GO:0006955˜immune response	7.34e-10
1	GO:0042110˜T cell activation	1.42e-07
2	GO:0006955˜immune response	4.03e-12
2	GO:0006414˜translational elongation	1.28e-08
2	hsa03010:Ribosome	2.96e-08
2	hsa04662:B cell receptor signaling pathway	4.31e-08
2	GO:0001775˜cell activation	2.00e-07
3	GO:0006414˜translational elongation	2.11e-86
3	hsa03010:Ribosome	3.28e-81
3	GO:0006412˜translation	6.62e-57
3	GO:0042273˜ribosomal large subunit biogenesis	3.27e-04
3	GO:0042254˜ribosome biogenesis	2.38e-03
4	GO:0006836˜neurotransmitter transport	8.83e-06
4	GO:0030182˜neuron differentiation	3.50e-03
4	GO:0007269˜neurotransmitter secretion	6.71e-03
4	GO:0050767˜regulation of neurogenesis	1.79e-02
4	GO:0048667˜cell morphogenesis involved in neuron diff.	1.83e-02
6	GO:0042110˜T cell activation	8.05e-11
6	hsa04660:T cell receptor signaling pathway	3.35e-09
6	GO:0045321˜leukocyte activation	1.08e-08
6	GO:0001775˜cell activation	1.43e-08
6	GO:0046649˜lymphocyte activation	2.44e-08
7	GO:0051276˜chromosome organization	3.86e-10
7	GO:0006350˜transcription	7.90e-10
7	GO:0006325˜chromatin organization	1.98e-09
7	GO:0045449˜regulation of transcription	4.23e-08
7	GO:0008380˜RNA splicing	1.88e-07
9	hsa04080:Neuroactive ligand-receptor interaction	1.03e-04
10	GO:0006955˜immune response	1.61e-23
10	hsa05330:Allograft rejection	7.40e-16
10	hsa04940:Type I diabetes mellitus	5.44e-15
10	hsa05332:Graft-versus-host disease	1.46e-12
10	hsa04672:Intestinal immune network for IgA production	3.05e-11

**Fig. 6:**
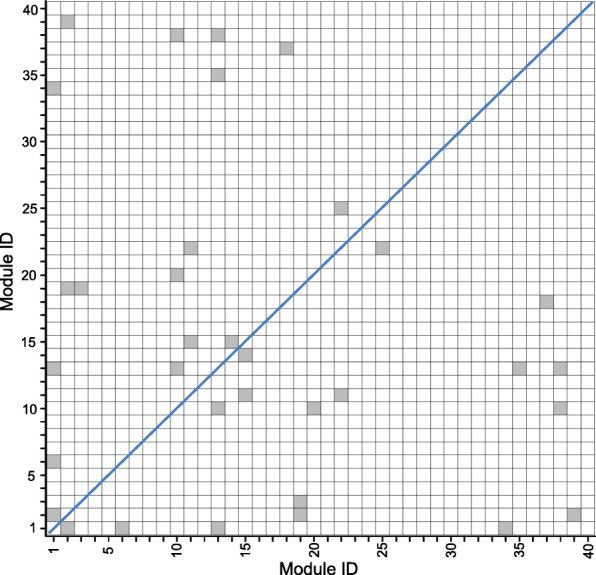
The overlapping significance level between any two identified modules by SNSVD on the CGP data. Each gray corresponds to a significance overlapping relationship (Hypergeometric test, *p* <0.05)

Finally, we also evaluated whether the identified 40 modules are greatly overlapped. Since each module contains a gene set and a sample set. To assess the overlapping relationship between two different modules. For any two gene modules, we computed the overlapping significance level *p*_1_ and *p*_2_ between their gene sets and sample sets respectively by using the right-tailed hypergeometric test. If *p*_1_<0.05 and *p*_2_<0.05, then we considered that the two modules are significant overlapped. Among all 780 module-module pairs for the identified 40 modules, we found that only 17 out of the 780 module-module pairs are significantly overlapped (Fig. 6), showing that our method can find diverse functional modules.

#### Comparison with sparse SVD on the CGP gene expression data sets

**Fig. 7:**
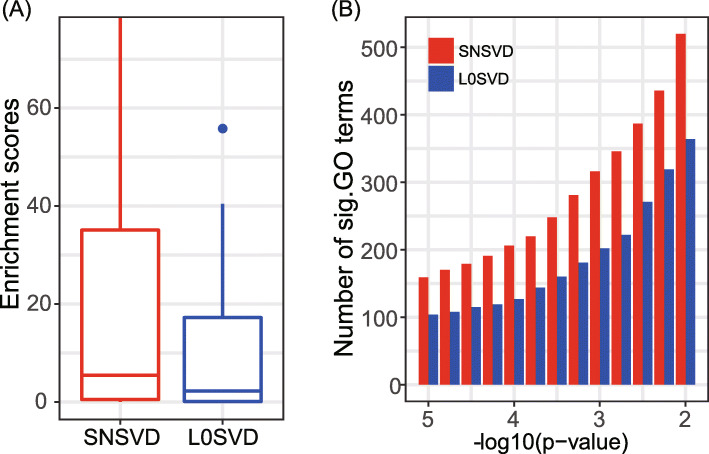
(A) Comparison of the gene-gene interaction enrichment scores of the identified modules by SNSVD and L0SVD, respectively, on the CGP data. (B) Functional enrichment comparison based on the number of GO BP (Gene Ontology Biological Process) terms

Since L0SVD have shown good performances in simulation study compared to other sparse SVD methods, we compared it with our method to further illustrate the importance of integrating the PPI network. To this end, we also identified 40 gene modules on the CGP data by using L0SVD and Fig. 7 shows the comparing results. We found that the interaction enrichment scores of the identified modules by SNSVD were significantly higher than that by L0SVD (one-sided Wilcoxon signed rank test *p*-value <0.01) (Fig. 7A). These results demonstrate that SNSVD can find more tightly connected genes than L0SVD by integrating the PPI network. Furthermore, SNSVD obtains a greater number of significant GO BP terms at different levels than L0SVD (one-sided Wilcoxon signed rank test *p*-value <0.001) (Fig. 7B), showing that incorporating the PPI network does help SNSVD to discover more biological interpretable modules.

### Application to the BRCA data sets

#### Data and preprocessing

**Fig. 8:**
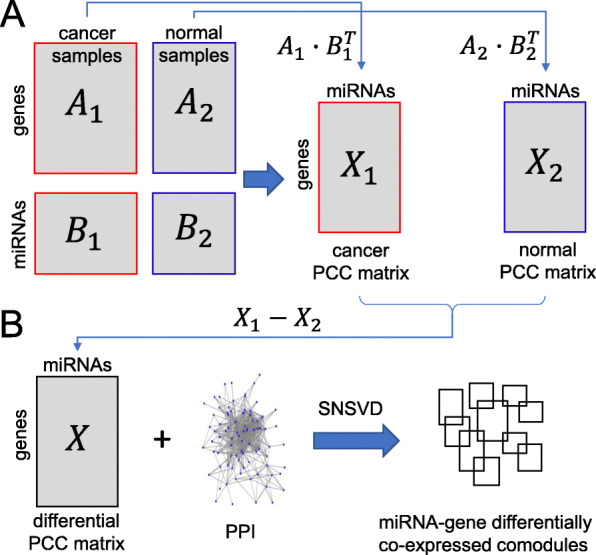
A framework based on SNSVD method for identifying differentially co-expressed miRNA-gene modules on the BRCA data. **A** Calculating two miRNA-gene correlation matrices ***X***_1_ and ***X***_2_ using the Pearson correlation method based on the four expression matrices ***A***_1_,***A***_2_,***B***_1_ and ***B***_2_ whose rows were centered and scaled. **B** We first obtain the differential correlation matrix ***X*** by using ***X***=***X***_1_−***X***_2_. Then, SNSVD is used to identify top ten differentially co-expressed miRNA-gene modules by integrating the differentially co-expressed matrix ***X*** and a PPI network

We downloaded the processed RNA-seq and miRNA-seq data of Breast invasive carcinoma (BRCA) from TCGA database [30] (Broad GDAC Firehose: http://firebrowse.org/). We firstly filtered out the genes and the miRNAs which are not expressed in more than 70% samples and the raw gene/miRNA expression values were log2-transformed. Secondly, we used the wilcoxon rank sum test to identify differentially expressed genes/miRNAs with bonferroni adjusted *p*-value <0.05 between cancer and adjacent normal samples. It causes 9896 differentially expressed genes and 320 differentially expressed miRNAs to be preserved. Thirdly, we imputed the missing values of miRNA and gene expression data by using k-nearest neighbors [31]. Finally, we extracted the matched gene and miRNA expression matrices across cancer and adjacent normal samples, where ***A***_1_ and ***B***_1_ represent gene and miRNA expression data of cancer samples, respectively and (ii) ***A***_2_ and ***B***_2_ represent gene and miRNA expression data of adjacent normal samples, respectively (Fig. 8A). There are 9896 genes and 320 miRNAs, 760 cancer samples and 87 adjacent normal samples in the BRCA data sets.

Additionally, we also downloaded a PPI network from Pathway-Commons database [26], and collected a set of cancer genes from the allOnco database (http://www.bushmanlab.org/links/genelists) which merges some different cancer genes from several databases, and a set of cancer miRNAs from the reference [32].

#### Identifying differentially co-expressed miRNA-gene modules

Recent research revealed that some abnormal miRNA-gene regulatory relationship plays key roles in tumor progression and development [33–35]. Some computational methods have been proposed for identifying miRNA-gene co-expressed modules by using matched miRNA and mRNA expression data of cancer [13, 36–40]. Though power, these methods do not ensure that the miRNAs and genes in a module are differentially expressed between two biological conditions. Besides, some methods have already been developed for differential co-expression analysis [41–44]. However, these methods only focus on single gene expression data analysis. To this end, we proposed a new framework based on SNSVD for analyzing matched miRNA and mRNA expression data between two biological conditions to identify differentially co-expressed miRNA-gene modules (Fig. 8).

Herein, we applied SNSVD to the BRCA data and empirically set *λ*,*k*_*v*_ in SNSVD to yield top ten differentially co-expressed modules for each *σ*. Each identified miRNA-gene module contain about 10 miRNAs and 100 genes. Formally, a miRNA-gene module contains a miRNA set and a gene set. We found that as *σ* becomes larger, the modules identified by SNSVD contain more edges (Table 2). The results showed SNSVD could overcome the drawbacks of sparse SVD (SNSVD with *σ*=0 in Table 2) to capture the modules with more edges by incorporating the PPI network.

**Table 2:** Application of SNSVD to the BRCA data. “edge.avg” represents the average of number of edges of modules in the PPI network, and “Fold Change” represents the fold change of “edge.avg” between the identified modules and random modules, and “d.avg” denotes the average of singular values of modules

Method	*σ*	edge.avg	FC.avg	d.avg
Sparse SVD	0	27.05	1.27	25.74
SNSVD	1	26.70	1.25	25.75
SNSVD	10	35.05	1.64	25.70
SNSVD	20	56.45	2.65	25.68
SNSVD	40	85.95	4.03	24.91
SNSVD	60	179.45	8.42	22.79
SNSVD	80	132.10	6.20	22.94
SNSVD	90	126.00	5.91	22.12
SNSVD	100	108.10	5.07	21.61
SNSVD	150	251.05	11.78	16.50
SNSVD	200	231.30	10.86	10.24

Note that SNSVD reduces to a sparse SVD when *σ*=0

#### Functional analysis of modules

**Fig. 9:**
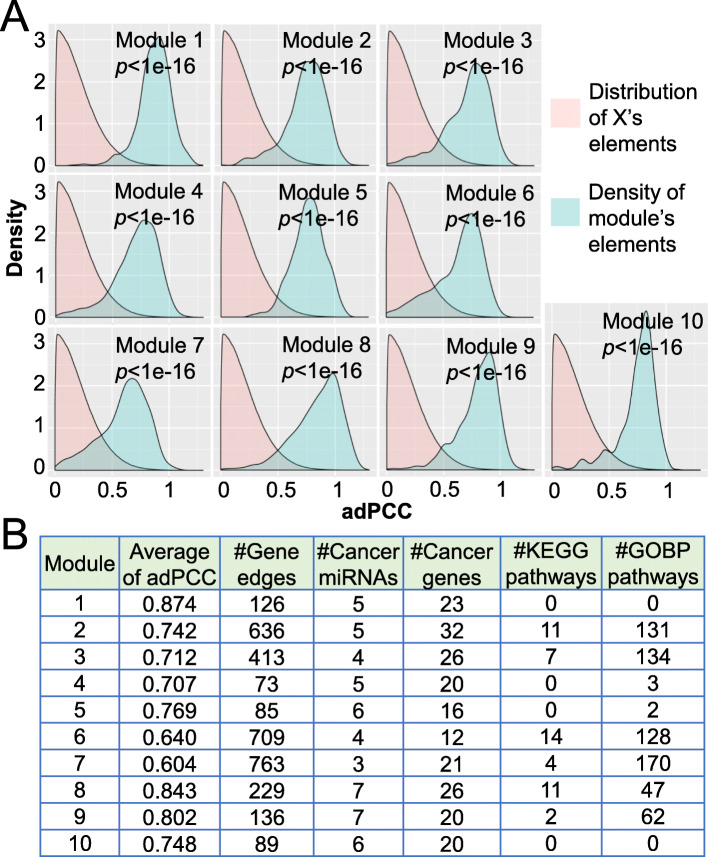
Biological function analysis of the identified ten differentially co-expressed miRNA-gene modules by SNSVD with *σ*=60. **A** For each module, the distribution (pink area) is fitted based on the absolute values of all the elements in the differentially co-expressed matrix ***X***, and the distribution (light blue area) is fitted based on the absolute values of the elements from the module corresponding submatrix in ***X***. *P*-values were computed by using permutation test. **B** For each module, “Average of adPCC” is the average of the absolute values of the elements from the module corresponding submatrix in ***X***. “#Gene edges”, “#Cancer gene”, “#Cancer miRNA”, “#KEGG pathways” and “#GOBP pathways” represent the number of interaction edges, cancer genes, cancer miRNAs, significantly enriched KEGG pathways and GO BP pathways (Benjamini-Hochberg adjusted *p*<0.05), respectively

Without loss of generality, the ten modules identified by SNSVD with *σ*=60 (See Table 2) were considered for further biological analysis. We found that (i) the average adPCC (absolute differential Pearson Correlation Coefficient) for the identified modules by SNSVD on the BRCA data is larger than the average of all absolute elements of ***X*** (Wilcoxon rank-sum test, *p*<1*e*−16) (Fig. 9A); (ii) more than half of the miRNAs in the 70% (7 of 10) modules are cancer miRNAs, and 80% (8 of 10) modules are significantly enriched at least one KEGG/GO BP pathway (Benjamini-Hochberg adjusted *p*<0.05); (iii) three modules (module 2, 3 and 8) contain significantly more cancer genes with hypergeometric test, *p*<0.05. More results are shown in Fig. 9B. Additionally, we obtained 39 miRNAs and 961 genes by combining the identified ten modules. We found that about 50% (19 of 39) miRNAs are cancer miRNAs, and about 21% (203 of 961) genes are cancer genes (hypergeometric test, *p*<4.3*e*−6).

## Discussion

In our previous work, SSVD has been developed for module discovery and its effectiveness has been demonstrated [13]. However, it cannot integrate the gene network data from PPI network. To this end, we develop the SNSVD method that integrates gene expression data and a gene interaction network to identify underlying gene functional modules. In the SNSVD, we define a sparse network regularized function which is a combination of *L*_1_-regularized norm and network-regularized norm to make the biclustering process tend to select interacted genes in the prior gene interaction network. Experimental results on the CGP and BRCA data demonstrate that SNSVD can overcome the drawbacks of SSVD. Although SNSVD is an effective method, some further studies are deserved to investigate: (1) extend SNSVD to identify non-linear relationships; (2) extend SNSVD to integrate other omics data, such as DNA methylation data; (3) apply SNSVD to other biological problems.

## Conclusions

In this paper, we presented a Sparse Network-regularized SVD (SNSVD) model for network-based cancer genomics data integration analysis and developed an alternating iterative algorithm to solve the model. By comparing with other representative methods on the simulated data and the real data, we found that SNSVD could find modules with high qualities by integrating the PPI interaction network. By investigating the modules identified by SNSVD on the CGP data, we found that all the genes within the same modules are co-expressed, and most genes in the same modules are connected with each other in the prior PPI network and enriched in at least one gene functional term. Besides, we also applied our method to the BRCA data from TCGA database for identifying ten differentially co-expressed miRNA-gene modules. Some breast cancer related miRNA-gene modules were discovered. To sum up, our work provides a promising way to integrate the network information into the sparse SVD framework, which can help to find biologically significant functional modules and makes the results easily interpreted. An R package of SNSVD is available at https://github.com/wenwenmin/SNSVD.

## Methods

### Sparse network-regularized SVD (SNSVD) model

Let $\boldsymbol {X}\in \mathbb {R}^{p\times n}$ (*p* genes and *n* samples) be the gene expression data. Suppose $\boldsymbol {A} \in \mathbb {R}^{p\times p}$ is an adjacency matrix of a PPI network, where ***A***_*ij*_=1 if vertex *i* and *j* is connected and ***A***_*ij*_=0 otherwise. Thus, the normalized Laplacian matrix ***L***=(***L***_*ij*_)_*p*×*p*_ encoding the PPI network can be defined as:

2 $$\begin{array}{@{}rcl@{}} \boldsymbol{L}_{{ij}}= \left\{\begin{array}{ll} 1, &\text{if}~i=j~\text{and}~d_{i}\neq 0,\cr -\frac{\boldsymbol{A}_{{ij}}}{\sqrt{d_{i}d_{j}}}, &\text{if}~i~\text{and}~j~\text{are~adjacent},\cr 0, &\text{otherwise}. \end{array}\right. \end{array} $$

where $d_{i} = \sum _{j=1}^{p} \boldsymbol {A}_{{ij}}$. Correspondingly, we have $\boldsymbol {u}^{T}\boldsymbol {L}\boldsymbol {u} = \frac {1}{2}\sum _{i}\sum _{j}\boldsymbol {A}_{{ij}}\left (\frac {u_{i}}{\sqrt {d_{i}}}-\frac {u_{j}}{\sqrt {d_{j}}}\right)^{2}$, which encourages the estimated coefficients of ***u*** to be smooth over adjacent genes in the PPI network ***A*** [20]. To further force ***u*** to be sparse, we introduce a sparse network-regularized penalty:

3 $$ P_{1}(\boldsymbol{u}) = \lambda \|\boldsymbol{u}\|_{1}+ \sigma \boldsymbol{u}^{T}\boldsymbol{L}\boldsymbol{u},  $$

where *λ* and *σ* are two parameters. In the penalty (3), the *L*_1_ norm (∥***u***∥_1_) is to induce sparsity in ***u***; and the quadratic Laplacian norm (***u***^*T*^***Lu***) makes the selected genes tend to connect with each other in the PPI network.

To integrate the network information in SVD framework, we present a sparse network-regularized SVD (SNSVD) model as follows:

4 $$ \begin{aligned} & \underset{\boldsymbol{u},\boldsymbol{v},d}{\text{minimize}} && \|\boldsymbol{X} - d\boldsymbol{uv}^{T}\|_{F}^{2}\\ & \text{subject to} && \|\boldsymbol{u}\|_{2}^{2} \leq 1, \lambda \|\boldsymbol{u}\|_{1} + \sigma \boldsymbol{u}^{T}\boldsymbol{L}\boldsymbol{u} \leq c_{1},\\ & && \|\boldsymbol{v}\|_{2}^{2} \leq 1, \|\boldsymbol{v}\|_{0} \leq k_{v}, \end{aligned}  $$

where *c*_1_ and *k*_*v*_ are two parameters to control the number of selected genes and samples separately. As for the samples, we simply use a *L*_0_-regularized penalty on ***v*** sample variables (corresponding to sample variables) to induce sparseness. Compared to *L*_1_-norm, *L*_0_-norm is known as the most essential sparsity measure and has nice theoretical properties [15, 45].

### SNSVD algorithm

Since $||\boldsymbol {X}-d\boldsymbol {u}\boldsymbol {v}^{T}||_{F}^{2} = tr\left (\boldsymbol {X}\boldsymbol {X}^{T}\right)+d^{2}tr\left (\boldsymbol {u}\boldsymbol {v}^{T}\boldsymbol {v}\boldsymbol {u}^{T}\right)-2d\boldsymbol {u}^{T}\boldsymbol {X}\boldsymbol {v}$, where *tr*(·) denotes the trace of a matrix; Both ***u*** and ***v*** are guaranteed to be two unit vectors, *tr*(***uv***^*T*^***vu***^*T*^)=*tr*(***u***^*T*^***uv***^*T*^***v***)=1. Minimizing $||\boldsymbol {X}-d\boldsymbol {u}\boldsymbol {v}^{T}||_{F}^{2}$ in Eq. (4) is equivalent to minimizing −***u***^*T*^***Xv***. Although there are three parameters ***u***,***v*** and *d* to be optimized in Eq. (4). It is notable that once ***u*** and ***v*** are fixed, then *d* can be determined *d*=***u***^*T*^***Xv*** in Eq. (4). Thus, to solve Eq. (4), we just need to optimize ***u*** and ***v***. Inspired by Ref. [46], we present an alternating iterative strategy to solve ***u*** and ***v***, i.e., fixing ***v*** to update ***u*** and fixing ***u*** to update ***v***.

Fixing ***v*** in Eq. (4), it is equivalent to solve the following sub-problem:

5 $$ \begin{aligned} & \underset{\boldsymbol{u}}{\text{minimize}} && {-\boldsymbol{u}^{T}\boldsymbol{X}\boldsymbol{v}}\\ & \text{subject to} && \|\boldsymbol{u}\|_{2}^{2} \leq 1, \lambda \|\boldsymbol{u}\|_{1}+ \sigma \boldsymbol{u}^{T}\boldsymbol{L}\boldsymbol{u} \leq c_{1}. \end{aligned}  $$

Let ***z***=***Xv***, the optimization problem in (5) can be redefined as follows:

6 $$ \begin{aligned} & \underset{\boldsymbol{u}}{\text{minimize}} && -\boldsymbol{u}^{T}\boldsymbol{z}\\ & \text{subject to} && \|\boldsymbol{u}\|_{2}^{2} \leq 1, \lambda \|\boldsymbol{u}\|_{1}+ \sigma \boldsymbol{u}^{T}\boldsymbol{L}\boldsymbol{u} \leq c_{1}. \end{aligned}  $$

To solve it, we write its Lagrangian form as follows:

7 $$ \mathcal{L}(\boldsymbol{u}) = -\boldsymbol{u}^{T}\boldsymbol{z}+ \eta \boldsymbol{u}^{T}\boldsymbol{u} + \lambda \|\boldsymbol{u}\|_{1} + \sigma \boldsymbol{u}^{T}\boldsymbol{L}\boldsymbol{u},  $$

where *λ*≥0, *η*≥0,*σ*≥0. In order to facilitate the calculation without loss of generality, we use $\frac {1}{2} \eta $ instead of $\eta, \frac {1}{2} \sigma $ instead of *σ*, then Eq. (7) can be rewritten as:

8 $$ \mathcal{L}(\boldsymbol{u}) = -\boldsymbol{u}^{T}\boldsymbol{z}+\frac{1}{2}\eta \boldsymbol{u}^{T}\boldsymbol{u} + \lambda \|\boldsymbol{u}\|_{1} + \frac{1}{2} \sigma \boldsymbol{u}^{T}\boldsymbol{L}\boldsymbol{u}.  $$

It is a convex function with respect to ***u***, therefore its optimal solution can be characterized by some sub-gradient equations (see e.g., [47]). Since ***L***=***I***−***D***^−1/2^***AD***^−1/2^ (based on Eq. 2). For convenience, let ***W***=***I***−***L***=***D***^−1/2^***AD***^−1/2^ (***D*** is a diagonal matrix and $\boldsymbol {D}_{{ii}} = \sum _{j} \boldsymbol {A}_{{ij}}$), then we have the sub-gradient equations of Eq. (8) as:

9 $$ \frac{\partial \mathcal{L}}{\partial \boldsymbol{u}_{j}} = -\boldsymbol{z}_{j} + \eta \boldsymbol{u}_{j} + \lambda s_{j} + \sigma \boldsymbol{u}_{j} -\sigma \boldsymbol{W}_{j}\boldsymbol{u} = 0,~j = 1,\cdots,p.  $$

where *s*_*j*_=sign(***u***_*j*_) if ***u***_*j*_≠0 and *s*_*j*_∈{*t*,|*t*|≤1} otherwise; and ***W***_*j*_ is the *j*th row of matrix ***W***. Let the solution of (8) be $ \widehat {\boldsymbol {u}}=\left (\widehat {\boldsymbol {u}}_{1}, \widehat {\boldsymbol {u}}_{2}, \cdots, \widehat {\boldsymbol {u}}_{p}\right)$. By using a coordinate descent method [48, 49], we obtain the following coordinate update rule for $\widehat {\boldsymbol {u}}_{j}$:

10 $$\begin{array}{@{}rcl@{}} \widehat{\boldsymbol{u}}_{j}= \left\{\begin{array}{ll} 0 &\text{if}~|\boldsymbol{z}_{j} + \sigma \boldsymbol{W}_{j}\widehat{\boldsymbol{u}}| \leq \lambda,\cr \frac{\boldsymbol{z}_{j} + \sigma \boldsymbol{W}_{j}\widehat{\boldsymbol{u}}- \lambda \text{sign}(\widehat{\boldsymbol{u}}_{j})}{\eta+\sigma} &\text{otherwise}. \end{array}\right. \end{array} $$

Define $\mathcal {S}(a,\lambda) = \text {sign}(a)(|a|-\lambda)_{+}$, we have $\widehat {\boldsymbol {u}}_{j} = \mathcal {S}(\boldsymbol {z}_{j} + \sigma \boldsymbol {W}_{j} \widehat {\boldsymbol {u}}, \lambda)/(\eta +\sigma)$. Let $\breve {\boldsymbol {u}}_{j} = \mathcal (\boldsymbol {z}_{j} + \sigma \boldsymbol {W}_{j}\widehat {\boldsymbol {u}}, \lambda)$ and $\breve {\boldsymbol {u}}=\left (\breve {\boldsymbol {u}}_{1},...,\breve {\boldsymbol {u}}_{p}\right)^{T}$, we can obtain a normalized solution $\boldsymbol {u}=\frac {\widehat {\boldsymbol {u}}}{\| \widehat {\boldsymbol {u}} \|_{2}}=\frac {\breve {\boldsymbol {u}}}{\| \breve {\boldsymbol {u}} \|_{2}}$. In a word, we use a coordinate descent method to minimize Eq. (8) and update one ***u***_*j*_ at a time while keeping ***u***_*k*_ fixed for all *k*≠*j*.

Fixing ***u*** in Eq. (4), it is equivalent to solve the following sub-problem:

11 $$ \begin{aligned} & \underset{\boldsymbol{v}}{\text{minimize}} && \|\boldsymbol{X} - d\boldsymbol{uv}^{T}\|_{F}^{2}\\ & \text{subject to} && \|\boldsymbol{v}\|_{2}^{2} \leq 1, \|\boldsymbol{v}\|_{0} \leq k_{v}. \end{aligned}  $$

Let $\boldsymbol {z}_{v} = \boldsymbol {X}^{T}\boldsymbol {u}, \boldsymbol {\widehat {v}} = d\boldsymbol {v}$, we thus have $\|\boldsymbol {X} - d\boldsymbol {uv}^{T}\|_{F}^{2} = \|\boldsymbol {z}_{v}-\boldsymbol {\widehat {v}}\|_{2}^{2} + c  $, where *c*=*tr*(***X***^*T*^***X***)−***u***^*T*^***XX***^*T*^***u***. Obviously *c* is a constant value with respect to ***v***. Thus problem (11) is equivalent to:

12 $$ \min_{\boldsymbol{\widehat{v}}} \|\boldsymbol{z}_{v} - \boldsymbol{\widehat{v}}\|_{2}^{2},~~\text{subject to}~\|\boldsymbol{\widehat{v}}\|_{0} \leq k_{v}.  $$

Its optimal solution is $\boldsymbol {\widehat {v}} = \boldsymbol {z}_{v} \bullet I(|\boldsymbol {z}_{v}| \geq |\boldsymbol {z}_{v}|_{(k_{v})})$ where *I*(·) is the indicator function and *"*∙*"* is point multiplication function, and |***z***_*v*_|_(*i*)_ denotes the *i*-th order statistic of |***z***_*v*_|, i.e. |***z***_*v*_|_(1)_≥|***z***_*v*_|_(2)_≥,...,≥|***z***_*v*_|_(*n*)_. In other words, we only keep the *k*_*v*_ variables of ***z***_*v*_ corresponding to its *k*_*v*_ largest absolute values. The normalized optimal solution of Eq. (11) is $\boldsymbol {v} = \widehat {\boldsymbol {v}}/\|\widehat {\boldsymbol {v}}\|_{2}$, i.e.,

13 $$ \boldsymbol{v} = \frac{\boldsymbol{z}_{v}\bullet I(|\boldsymbol{z}_{v}| \geq |\boldsymbol{z}_{v}|_{(k_{v})})}{\|\boldsymbol{z}_{v}\bullet I(|\boldsymbol{z}_{v}| \geq |\boldsymbol{z}_{v}|_{(k_{v})})\|_{2}}.  $$

Finally, we develop an alternating iterative algorithm by alternately updating ***u*** and ***v*** to solve SNSVD model. The details of this algorithm is given in Algorithm 1, and its time complexity is $\mathcal {O}\left (Tnp + Tp^{2} + Tn^{2}\right)$, where *T* is the number of iterations.


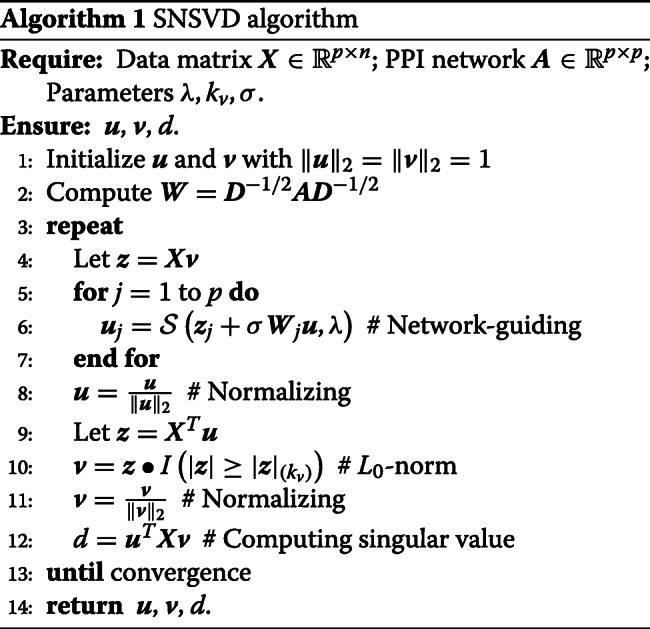


### Convergence analysis of SNSVD algorithm

Next, we give the convergence analysis of Algorithm 1. In fact, Algorithm 1 is to solve the Lagrangian form of problem (4) as follows:

14 $$ \begin{aligned} & \underset{\boldsymbol{u},\boldsymbol{v},d}{\text{minimize}} && -\boldsymbol{u}^{T}\boldsymbol{X}\boldsymbol{v} + \lambda \|\boldsymbol{u}\|_{1} + \sigma \boldsymbol{u}^{T}\boldsymbol{L}\boldsymbol{u}\\ & \text{subject to} && \|\boldsymbol{u}\|_{2}^{2} \leq 1, \|\boldsymbol{v}\|_{2}^{2} \leq 1, \|\boldsymbol{v}\|_{0} \leq k_{v}. \end{aligned}  $$

Let *H*(***u***,***v***)=−***u***^*T*^***Xv***+*σ****u***^*T*^***Lu***,*f*(***u***)=*ρ*(***u***)+*λ*∥***u***∥_1_ and *g*(***v***)=*ρ*(***v***)+*τ*(***v***,*k*_*v*_) where

15a $$\begin{array}{*{20}l} \rho(\boldsymbol{u}) & = \left\{\begin{array}{ll} 0, &\text{if}~\|\boldsymbol{u}\|_{2}^{2} \leq 1\cr +\infty, &\text{otherwise}. \end{array}\right. \end{array} $$

15b $$\begin{array}{*{20}l} \rho(\boldsymbol{v}) & = \left\{\begin{array}{ll} 0, &\text{if}~\|\boldsymbol{v}\|_{2}^{2} \leq 1\cr +\infty, &\text{otherwise}. \end{array}\right. \end{array} $$

15c $$\begin{array}{*{20}l} \tau(\boldsymbol{v},k_{v}) & = \left\{\begin{array}{ll} 0,&\text{if}~\|\boldsymbol{v}\|_{0}\leq k_{v}\cr +\infty,&\text{otherwise}. \end{array}\right. \end{array} $$

Therefore the Lagrangian form of problem (4) can be written as *F*(***u***,***v***)=*H*(***u***,***v***)+*f*(***u***)+*g*(***v***) which is a semialgebraic function [46]. Based on the Theorem 1 in [46], Algorithm 1 converges to a critical point of *F*(***u***,***v***).


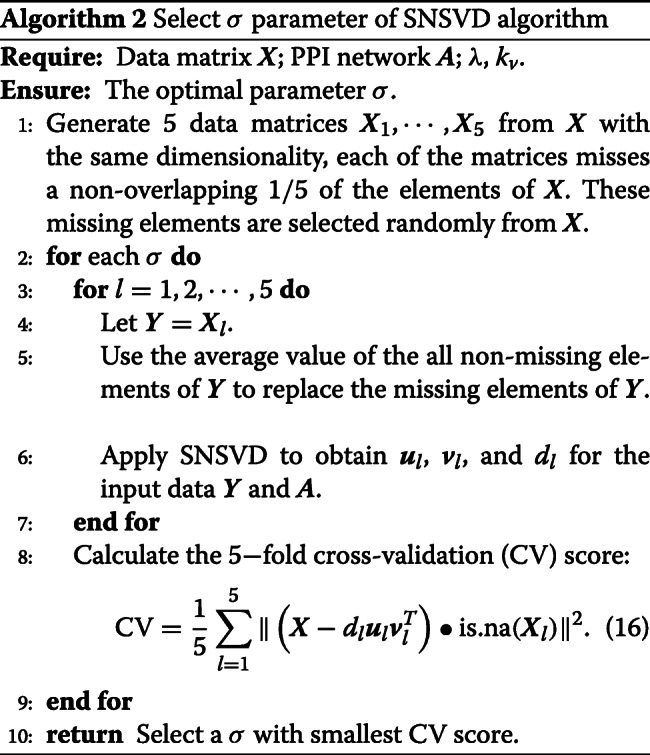


### Parameter selection of SNSVD algorithm

As to *λ* and *k*_*v*_’s choice in Algorithm 1 when it is applied to the CGP gene expression data, we select a suitable *λ* to force the estimated ***u*** only containing 200 nonzero elements which is beneficial for further analysis of the biological function of the module and set *k*_*v*_=50 (control the sample sparsity) which ensures that the number of samples within in the module is approximately the same as the number of samples of a subtype. As to *σ*’s choice in Algorithm 1, we present a 5-fold cross-validation framework (Algorithm 2). To this end, we define a binary matrix is.na(***X***) with the same size of ***X*** and the elements are 1 if they are missing in ***X***, 0 otherwise.

### Learning multiple pairs of singular vectors using SNSVD

It is notable that every run of Algorithm 1 can only obtain a pair of sparse singular vectors ***u*** and ***v*** (Fig. 1). In order to identify multiple modules, we can repeat running Algorithm 1. After each turn of the iteration, we use the obtained ***u***,***v*** and *d* to modify the gene expression data ***X***, (***X***:=***X***−*d****uv***^*T*^), the modified ***X*** is then used as the new input data for the next run to obtain the next pair of singular vectors. Moreover, we notice that Algorithm 1 may get different local optima with different initials, we run Algorithm 1 five times with different initials which are generated according to the multivariate standard normal distribution and choose the best one as the final solution of each turn. The detailed procedure is described in Algorithm 3.


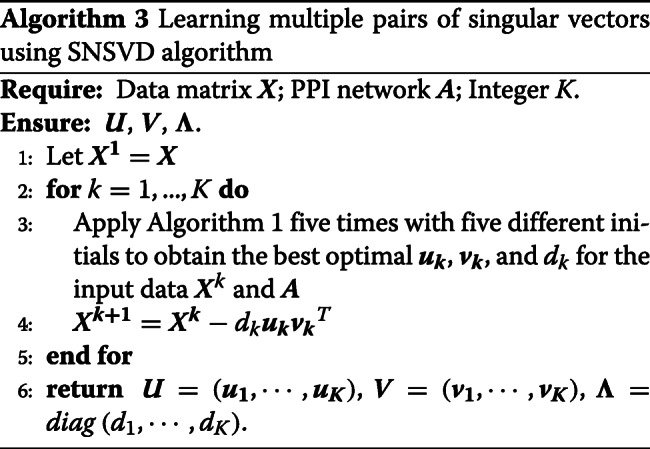


### Modularity score

To assess whether the genes within the same module are co-expressed/correlated, we use a *modularity* score to describe the overall co-expression of genes within the module. For a given module *k* containing *p*_*k*_ genes and *n*_*k*_ samples, we first calculate the correlation between gene *i* and *j* across the *n*_*k*_ samples, denoted as *w*_*ij*_. For convenience, we force to set *w*_*ii*_=0 for each *i*. Then the *modularity* score of the module can be defined as:

16 $$ \text{Modularity} = \frac{1}{p_{k}\cdot(p_{k}-1)} \sum\limits_{i=1}^{p_{k}} \sum\limits_{j=1}^{p_{k}} |w_{{ij}}|.  $$

Intuitively, if a module has a high modularity score, then the genes within the module is highly co-expressed.

### Gene-gene interaction enrichment score

In order to evaluate whether the genes within the same module are tightly connected in the prior PPI network, we use the right tailed hypergeometric test to compute a significance level of each module. Suppose that the PPI network contains *n* genes and *m* edges, and module *i* contains *n*_*i*_ genes and *m*_*i*_ edges, then the significance level of module *k* can be calculated via the following equation:

17 $$ p(i) = \sum_{x< m_{i}}\frac{\binom{m}{x} \binom{N-m}{m_{i}-x}}{\binom{N}{N_{i}}},  $$

where $N = \binom {n}{2}$ and $N_{i} = \binom {n_{i}}{2}$. Accordingly, we can define the gene-gene interaction enrichment score *s*(*i*) of the module *i* by the following formula:

18 $$ s(i) = -\text{log}_{10}(p(i)).  $$

The higher the gene-gene interaction enrichment score is, the denser the genes connect with each other. If the score is higher than 1.3, then the genes are significantly inter-connected with each other in the PPI network.

## Data Availability

The CGP datasets used during the current study are available in the Cancer Genome Project (CGP) repository (http://www.cancerrxgene.org/downloads). And the BRCA datasets used during the current study are available in the TCGA repository (Broad GDAC Firehose: http://firebrowse.org/).

## Abbreviations

SVD: Singular value decomposition
SSVD: Sparse singular value decomposition
SNSVD: Sparse network-regularized singular value decomposition
LASSO: Least absolute shrinkage and selection operator
SNR: Signal-to-noise ratio
CGP: Cancer genome project
TCGA: The cancer genome atlas
PPI: Protein protein interaction
KEGG: Kyoto encyclopedia of genes and genomes
GO: Gene ontology

## References

[1] Wan Q, Dingerdissen H, Fan Y, Gulzar N, Pan Y, Wu TJ, Yan C, Zhang H, Mazumder R (2015). Bioxpress: An integrated RNA-seq-derived gene expression database for pan-cancer analysis. Database (Oxford).

[2] Vallejos CA, Risso D, Scialdone A, Dudoit S, Marioni JC (2017). Normalizing single-cell RNA sequencing data: challenges and opportunities. Nat Methods.

[3] Iorio F, Knijnenburg TA, Vis DJ, Bignell GR, Menden MP, Schubert M, Aben N, Gonçalves E, Barthorpe S, Lightfoot H (2016). A landscape of pharmacogenomic interactions in cancer. Cell.

[4] Lee M, Shen H, Huang JZ, Marron J (2010). Biclustering via sparse singular value decomposition. Biometrics.

[5] Liquet B, de Micheaux PL, Hejblum BP, Thiébaut R (2015). Group and sparse group partial least square approaches applied in genomics context. Bioinformatics.

[6] Min W, Liu J, Zhang S (2018). Edge-group sparse pca for network-guided high dimensional data analysis. Bioinformatics.

[7] Liu X, Chang X, Liu R, Yu X, Chen L, Aihara K (2017). Quantifying critical states of complex diseases using single-sample dynamic network biomarkers. PLoS Comput Biol.

[8] Yu X, Zhang J, Sun S, Zhou X, Zeng T, Chen L (2017). Individual-specific edge-network analysis for disease prediction. Nucleic Acids Res.

[9] Eren K, Deveci M, Küçüktunç O, Ümit V (2013). Çatalyürek: A comparative analysis of biclustering algorithms for gene expression data. Brief Bioinforma.

[10] Sill M, Kaiser S, Benner A, Kopp-Schneider A (2011). Robust biclustering by sparse singular value decomposition incorporating stability selection. Bioinformatics.

[11] Oghabian A, Kilpinen S, Hautaniemi S, Czeizler E. Biclustering methods: Biological relevance and application in gene expression analysis. PLoS ONE. 2014; 9(3). 10.1371/journal.pone.0090801 PMC3961251 24651574

[12] Chen S, Liu J, Zeng T (2015). Measuring the quality of linear patterns in biclusters. Methods.

[13] Min W, Liu J, Luo F, Zhang S (2016). A two-stage method to identify joint modules from matched microRNA and mRNA expression data. IEEE Trans Nanobiosci.

[14] Yang D, Ma Z, Buja A (2016). Rate optimal denoising of simultaneously sparse and low rank matrices. J Mach Learn Res.

[15] Asteris M, Kyrillidis A, Koyejo O, Poldrack R. A simple and provable algorithm for sparse diagonal CCA. In: International Conference on Machine Learning: 2016. p. 1148–1157.

[16] Sokolov A, Carlin DE, Paull EO, Baertsch R, Stuart JM (2016). Pathway-based genomics prediction using generalized elastic net. PLoS Comput Biol.

[17] Hill SM, Heiser LM (2016). Inferring causal molecular networks: empirical assessment through a community-based effort. Nat Methods.

[18] Enrico G (2016). Using prior knowledge from cellular pathways and molecular networks for diagnostic specimen classification. Brief Bioinforma.

[19] Lee E, Chuang H-Y, Kim J-W, Ideker T, Lee D (2008). Inferring pathway activity toward precise disease classification. PLoS Comput Biol.

[20] Li C, Li H (2008). Network-constrained regularization and variable selection for analysis of genomic data. Bioinformatics.

[21] Sun H, Feng R, Lin W, Li H (2013). Network-regularized high-dimensional cox regression for analysis of genomic data. Stat Sin.

[22] Chen J, Zhang S (2016). Integrative analysis for identifying joint modular patterns of gene-expression and drug-response data. Bioinformatics.

[23] Zhu F, Liu J, Min W. Gene functional module discovery via integrating gene expression and ppi network data. In: International Conference on Intelligent Computing: 2019. p. 116–126. 10.1007/978-3-030-26969-2_11.

[24] Tibshirani R (1996). Regression shrinkage and selection via the lasso. J R Stat Soc Ser B (Methodological).

[25] Fan J, Li R (2001). Variable selection via nonconcave penalized likelihood and its oracle properties. J Am Stat Assoc.

[26] Cerami EG, Gross BE (2011). Pathway commons, a web resource for biological pathway data. Nucleic Acids Res.

[27] Huang DW, Sherman BT, Lempicki RA (2009). Systematic and integrative analysis of large gene lists using DAVID bioinformatics resources. Nat Protocol.

[28] Leeksma OC, de Miranda NF, Veelken H (2017). Germline mutations predisposing to diffuse large B-cell lymphoma. Blood Cancer J.

[29] Disis ML (2010). Immune regulation of cancer. J Clin Oncol.

[30] Lander ES, Park PJ (2013). The cancer genome atlas pan-cancer analysis project. Nat Genet.

[31] Troyanskaya O, Cantor M (2001). Missing value estimation methods for DNA microarrays. Bioinformatics.

[32] Xie B, Ding Q, Han H, Wu D (2013). miRCancer: a microRNA cancer association database constructed by text mining on literature. Bioinformatics.

[33] Garzon R, Calin GA, Croce CM (2009). MicroRNAs in cancer. Ann Rev Med.

[34] Adams BD, Kasinski AL, Slack FJ (2014). Aberrant regulation and function of microRNAs in cancer. Curr Biol.

[35] Iorio MV, Croce CM (2009). MicroRNAs in cancer: small molecules with a huge impact. J Clin Oncol.

[36] Zhang S, Li Q, Liu J, Zhou XJ (2011). A novel computational framework for simultaneous integration of multiple types of genomic data to identify microRNA-gene regulatory modules. Bioinformatics.

[37] Zhang S, Liu C-C, Li W, Shen H, Laird PW, Zhou XJ (2012). Discovery of multi-dimensional modules by integrative analysis of cancer genomic data. Nucleic Acids Res.

[38] Bryan K (2013). Discovery and visualization of miRNA-mRNA functional modules within integrated data using bicluster analysis. Nucleic Acids Res.

[39] Li Y, Liang C, Wong K-C, Luo J, Zhang Z (2014). Mirsynergy: detecting synergistic miRNA regulatory modules by overlapping neighbourhood expansion. Bioinformatics.

[40] Jin D, Lee H (2015). A computational approach to identifying gene-microRNA modules in cancer. PLoS Comput Biol.

[41] Tesson BM, Breitling R, Jansen RC (2010). DiffCoEx: a simple and sensitive method to find differentially coexpressed gene modules. BMC Bioinformatics.

[42] Ideker T, Krogan NJ (2012). Differential network biology. Mol Syst Biol.

[43] Ha MJ, Baladandayuthapani V, Do K-A (2015). Dingo: differential network analysis in genomics. Bioinformatics.

[44] Zhu L (2016). MetaDCN: meta-analysis framework for differential co-expression network detection with an application in breast cancer. Bioinformatics.

[45] Yang F, Shen Y, Liu ZS (2017). The proximal alternating iterative hard thresholding method for *L*_0_ minimization, with complexity $\mathcal {O}(1/\sqrt {k})$. J Comput Appl Math.

[46] Bolte J, Sabach S, Teboulle M (2014). Proximal alternating linearized minimization for nonconvex and nonsmooth problems. Math Program.

[47] Nesterov Y (2009). Primal-dual subgradient methods for convex problems. Math Program.

[48] Friedman J, Hastie T, Höfling H, Tibshirani R (2007). Pathwise coordinate optimization. Ann Appl Stat.

[49] Friedman J, Hastie T, Tibshirani R (2010). Regularization paths for generalized linear models via coordinate descent. J Stat Softw.

